# Draft Genome Sequence of *Vibrio mimicus* Strain CAIM 602^T^

**DOI:** 10.1128/genomeA.00084-13

**Published:** 2013-03-14

**Authors:** Iliana Guardiola-Avila, Evelia Acedo-Felix, Lorena Noriega-Orozco, Gloria Yepiz-Plascencia, Itzel Sifuentes-Romero, Bruno Gomez-Gil

**Affiliations:** a CIAD (Research Center for Food and Development), Hermosillo, Sonora, Mexico; b CIAD, Guaymas Unit—Quality Assurance and Management of Natural Resources, Guaymas, Sonora, Mexico; c CIAD Mazatlán Unit for Aquaculture and Environmental Management, Mazatlán, Sinaloa, Mexico

## Abstract

*Vibrio mimicus* is a Gram-negative bacterium associated with gastrointestinal diseases in humans around the world. We report the complete genome sequence of the *Vibrio mimicus* strain CAIM 602^T^ (CDC1721-77, LMG 7896^T^, ATCC 33653^T^).

## GENOME ANNOUNCEMENT

*Vibrio mimicus* can cause gastrointestinal diseases in humans (1). It has been found to be associated with aquatic environments, such as fresh, brackish, and seawater (2). Studies around the world (in locations including Bangladesh, Japan, Costa Rica, Mexico, Thailand, the United States, Nigeria, Brazil, and Australia, among others) have demonstrated the presence of *V. mimicus* in foods, water samples, sediments, and aquatic plants (1–5).

Like other *Vibrio* species, *V. mimicus* has two chromosomes of different sizes, the larger of 3.0 Mb and a smaller of 1.5 Mb (6). The larger chromosome carries essential genes for growth and viability, as well as many virulence genes, while the smaller is where genes for adaptation to environmental change and a variant of *Vibrio* pathogenicity island 2 (VPI-2) are localized (7).

The type strain of *V. mimicus* was isolated from a 35-year-old female in North Carolina and was first designated a type strain in 1981 (5). It has been used as a control strain around the world and is partially sequenced (housekeeping genes).

CAIM 602^T^ was sequenced using a semiconductor NGS platform (Ion Torrent Personal Genome Machine sequencer; Life Technologies) with a 316 chip at CIAD Mazatlán. A total of 768,833 reads were obtained (mean length, 136 bp) for a total of 101 Mb and coverage of 23.5×; 611,888 reads were assembled with MIRA version 3.1.0 to obtain 120 contigs (*N*_50_, 159,054 bp). A genome-wide assembly and contig synteny was constructed with Mauve genome alignment software version 2.3.1 using *V. cholerae* N16961 as a reference strain (8). The contigs were further reassembled with Geneious R6 version 6.0.3 (Biomatters Ltd.) and refined with Gap4 version 2.0.0b9 of the Staden package (9) to obtain 11 supercontigs in chromosome I (ChI) and 5 in chromosome II (ChII) and 37 contigs in ChII as part of the superintegron. Original contigs were annotated by RAST (10) (http://rast.nmpdr.org/), where 55% of the features found were classified within a subsystem.

The assembled genome of the type strain *V. mimicus* CAIM 602 is 4.30 Mb in size and has two chromosomes. Chromosome I is approximately 2.90 Mb, with six rRNA operons, 71 tRNAs, 2,639 coding sequences (CDS), and a G+C content of 46.6%. Chromosome II is approximately 1.40 Mb; it has six tRNAs, 1,383 CDS, no rRNAs, and a G+C content of 46.2%. No plasmids were detected. The chromosome I sequence revealed various genes that encode virulence factors (e.g., hemolysins, proteases, mannose-sensitive hemagglutinin [MSHA] pili, type II and IV secretion systems, a putative RTX toxin, an aerobactin siderophore receptor, capsular polysaccharide biosynthesis protein, and an accessory colonization factor). Also revealed were several hypothetical proteins and genes with roles in DNA and RNA metabolism. Chromosome II contains genes encoding several membrane proteins and various hypothetical proteins, genes involved in cell signaling (genes for CdgA and GGDEF family proteins), genes encoding transcriptional regulator LysR family and AraC family proteins, multidrug resistance proteins, and type VI secretion proteins (VasI, VasL), and a lipoprotein ToxR-activated gene. A superintegron of at least 260 kb with approximately 402 CDS was found in ChII. One 13.2-kb prophage was also detected.

### Nucleotide sequence accession numbers.

*Vibrio mimicus* CAIM 602^T^ was obtained from the Collection of Aquatic Important Microorganisms (CAIM) (http://www.ciad.mx/caim). This whole-genome shotgun project has been deposited at DDBJ/EMBL/GenBank under the accession number AOMO00000000. The version described in this paper is the first version, AOMO01000000.

## References

[1] Chitov T Kirikaew P Yungyune P Ruengprapan N Sontikun K 2009 An incidence of large foodborne outbreak associated with *Vibrio mimicus*. Eur. J. Clin. Microbiol. Infect. Dis. 28:421–424 18925423 10.1007/s10096-008-0639-7

[2] Vieira VV Teixeira L Vicente AC Momen H Salles CA 2001 Differentiation of environmental and clinical isolates of *Vibrio mimicus* from *Vibrio cholerae* by multilocus enzyme electrophoresis. Appl. Environ. Microbiol. 67:2360–2364 11319123 10.1128/AEM.67.5.2360-2364.2001 PMC92878

[3] Adeleye IA Daniels FV Enyinnia VA 2010 Characterization and pathogenicity of *Vibrio spp*. contaminating seafoods in Lagos, Nigeria. Internet J. Food Saf. 12:1–9 http://www.internetjfs.org/articles/ijfsv12-1.pdf

[4] Begum A Khan SI 2001 Abundance of *Vibrio cholerae* non-O1 and *Vibrio mimicus* in various aquatic components of the pod ecosystem in Matlab, Bangladesh. Microbes Environ. 16:19–23

[5] Davis BR Fanning R Madden JM Steigerwalt AG Bradford HB Smith HL Brenner DJ 1981 Characterization of biochemically atypical *Vibrio cholerae* Strains and designation of a new pathogenic species, *Vibrio mimicus*. J. Clin. Microbiol. 14:631–639 7037833 10.1128/jcm.14.6.631-639.1981 PMC274012

[6] Okada K Iida T Kita-Tsukamoto K Honda T 2005 Vibrios commonly possess two chromosomes. J. Bacteriol. 187:752–757 15629946 10.1128/JB.187.2.752-757.2005 PMC543535

[7] Hasan NA Grim CJ Haley BJ Chun J Alam M Taviani E Hoq M Munk AC Saunders E Brettin TS Bruce DC Challacombe JF Detter JC Han CS Xie G Huq A Colwell R Colwell RR 2010 Comparative genomics of clinical and environmental *Vibrio mimicus*. Proc. Natl. Acad. Sci. U. S. A. 107:21134–21139 doi:10.1073/pnas.1013825107 21078967 10.1073/pnas.1013825107 PMC3000290

[8] Darling AC Mau B Blattner FR Perna NT 2004 Mauve: multiple alignment of conserved genomic sequence with rearrangements. Genome Res. 14:1394–1403 http://genome.cshlp.org/content/14/7/1394.full.pdf+html 15231754 10.1101/gr.2289704 PMC442156

[9] Staden R Beal KF Bonfield JK 2000 The Staden package. Methods Mol. Biol. 1998:115–130 10547834 10.1385/1-59259-192-2:115

[10] Aziz RK Bartels D Best AA DeJongh M Disz T Edwards RA Formsma K Gerdes S Glass EM Kubal M Meyer F Olsen GJ Olson R Osterman AL Overbeek RA McNeil LK Paarmann D Paczian T Parrello B Pusch GD Reich C Stevens R Vassieva O Vonstein V Wilke A Zagnitko O 2008 The RAST Server: rapid annotations using subsystems technology. BMC Genomics 9:75 18261238 10.1186/1471-2164-9-75 PMC2265698

